# The impact of behaviour change communication on the use of insecticide treated nets: a secondary analysis of ten post-campaign surveys from Nigeria

**DOI:** 10.1186/s12936-016-1463-7

**Published:** 2016-08-19

**Authors:** Albert Kilian, Harriet Lawford, Chinazo N. Ujuju, Tarekegn A. Abeku, Ernest Nwokolo, Festus Okoh, Ebenezer Baba

**Affiliations:** 1 Tropical Health LLP, Montagut, Spain; 2 Society for Family Health, Abuja, Nigeria; 3 Malaria Consortium, London, UK; 4 National Malaria Elimination Programme, Abuja, Nigeria; 5 Malaria Consortium Nigeria Office, Abuja, Nigeria

**Keywords:** Malaria, Nigeria, ITN use, IEC/BCC

## Abstract

**Background:**

Mass distribution campaigns of insecticide-treated nets for malaria prevention are usually accompanied by intensive behaviour change communication (BCC) to encourage hanging and use of nets. However, data on the effectiveness of these communication efforts are scarce. In preparation for the next round of mass campaigns in Nigeria, a secondary analysis of existing data from post-campaign surveys was undertaken to investigate the influence of BCC on net hanging and use.

**Methods:**

Surveys were undertaken between 2009 and 2012 in ten states in Nigeria using standardized questionnaires. Two-stage cluster sampling was used to select households in each study site. Outcomes were defined as the effects of BCC message exposure and recall on knowledge, attitudes, perception as well as intentions and actual use. From the univariable analysis, potential confounders and explanatory variables were identified and key effects explored in multivariable linear or logistic regression models; terms in the models were kept if they had a marginal significance with *p* < 0.2. To quantify the effects from BCC, a treatment effect model was used with an inverse-probability weight regression adjustment.

**Results:**

More than half of the respondents (58.4 %; 95 % CI 56.0, 60.7) had heard a message about net use or hanging during or after the distribution campaign, with media cited as the most common source of information. Attitude towards net use was positively linked to the number of messages recalled and was overall better in the northern study sites. The number of messages recalled was also the strongest predictor of knowledge (*p* < 0.001). All BCC outcomes showed a significant increase in net use, which was strongest for the confidence to take action regarding nets with an overall effect of 17 %-point increase of net use comparing poor and excellent confidence levels. Intention to use every night increased net use by 15 %-points and discussing net use in the family by 8 % points. All these effects were statistically significant (*p* < 0.001).

**Conclusions:**

Multichannel BCC campaigns as well as other media were effective in contributing to an increase in net culture, hanging and use, particularly by vulnerable groups.

**Electronic supplementary material:**

The online version of this article (doi:10.1186/s12936-016-1463-7) contains supplementary material, which is available to authorized users.

## Background

Over the past 10 years, the population in sub-Saharan Africa with access to insecticide-treated nets (ITNs) has increased significantly from around 3 % in 2004 to 49 % in 2014 [1]. Intensified malaria control campaigns and use of malaria control tools have contributed to a significant decrease in malaria morbidity rates, with rates falling by 47 % worldwide, and 54 % in the World Health Organization (WHO) Africa region, since 2000 [1]. Consistent use of ITNs can reduce malaria transmission by up to 90 % and avert as much as 44 % of all-cause mortality among children under 5 years of age [2, 3]. Mosquito nets are considered a useful predictor of epidemiological impact [4], and in efficacy studies, ITN use is a proxy for final health outcomes (reduction in malaria cases and deaths) [2, 5]. Studies have shown a community-level protective effect in areas with high ITN coverage, even if not all individuals use an ITN, wherein there is a community-effect resulting reduction in overall malaria transmission [6, 7]. Given the effectiveness of ITNs, the WHO recommended universal coverage of long-lasting insecticidal nets (LLINs) in 2007, defined as one LLIN for every two persons in all households within the target area [8].

One challenge when implementing universal coverage net distribution is ensuring that ITNs/LLINs are used consistently [9]; information, education and communication (IEC) and behaviour change communication (BCC) interventions are often used during or following mass distribution campaigns to encourage the correct hanging and use of ITNs [10]. Nonetheless, continual evidence has shown that despite increasing coverage and ownership of ITNs, net utilization is not synonymous with ownership [11]. This gap between ITN ownership and use has been attributed to an inability or unwillingness to hang and/or use ITNs [6, 9, 12–15] or a failure of BCC programmes to convince people to use available nets [4]. However, accessibility to ITN by all household members has been found to be the most important determinant of ITN use; in Papua New Guinea, it was found that 99.5 % of household members not using a net did not have access to one within the household [16], and a review of ITN use among children in 15 sub-Saharan African countries found intra-household access to ITNs to be the strongest and most consistent determinant of use among children [13]. Therefore, it is essential to define the ‘use gap’ [17] and differentiate between ‘non-use’ due to insufficient numbers of, or unusable, ITN within the household, in which further distribution is required, and the behavioural failure to use an ITN even though it is available, indicating the need for BCC to encourage net use [10, 13, 16].

Qualitative studies have suggested (within the limitations inherent in such studies) multiple reasons for not using an ITN, unrelated to access and availability [11, 18] and reasons should be expected to vary by locality, thus subsequent interventions need to be based on local evidence of why people do not use mosquito nets [16] as well as sound concepts of how use behaviour can be influenced [19]. Understanding why available ITNs are not used is needed to develop targeted IEC/BCC activities that can ultimately contribute to reducing malaria morbidity and mortality [18] and ensure more targeted and cost-effective strategies are developed [10]. The widespread use of BCC approaches for promoting ITNs is hampered by continued scepticism regarding their effectiveness [20], despite a previous study in Zambia suggesting that BCC “are necessary to improve use” and could “contribute towards closing the gap between ownership and use” [14].

Nigeria is the most populous country in Africa and 100 % of the population lives in an area of high transmission for malaria [1]. Malaria accounts for 60 % of outpatient visits, 30 % of hospitalizations and is the leading cause of mortality in children under 5 years old [21]; in 2013, Nigeria reported 37 million malaria cases accounting for 29 % of the total estimated malaria cases in sub-Saharan Africa [1]. Nigeria’s National Malaria Elimination Programme (NMEP) started the first round of mass campaigns for the distribution of LLINs to all households in 2009 in Kano State [22] and targeted two LLINs for every household registered by the mobilization teams. ITN ownership has increased from less than 10 % pre-campaigns to 50 % in 2013 [24]. Overall, 36 % of households have access to an ITN, and 24 % of the household population in households with at least one ITN slept under an ITN the night before the survey [23]. Since continuous distribution systems are still in their infancy in many parts of Nigeria, NMEP and partners are currently preparing for a new round of mass distributions of LLIN in order to further increase LLIN ownership and reach universal coverage. LLIN are also provided at highly subsidized rates through social marketing in health facilities and drug stores for ease of access to pregnant women and women with children under 5 years [24].

In preparation of the new LLIN distribution exercise, efforts were undertaken to identify shortcomings of the previous distribution campaign in order to optimize procedures for implementation and follow-up. This included a literature review on aspects of net and ITN use in Nigeria, which identified a difference in net culture between different parts of the country but also a lack of understanding of the determinants of net hanging and use and how these can best be influenced by BCC activities. As one of the outcomes of the review, two immediate operational research activities were suggested to inform the implementation of the campaign roll-out: (i) a mapping of BCC activities by all partners and stakeholders in the recent past with respect to messages, channels, content, intensity, and quality of messaging; and, (ii) a secondary analysis of existing data from post-campaign surveys that had been undertaken by different projects to assess the campaign outcomes and which included detailed information on net hanging and use as well as information of BCC exposure and message recall. Examples of IEC/BCC materials on LLIN use that were used by stakeholders included television and radio jingles and documentaries, audio songs, radio drama and magazines, billboards, brochures and toolkits, calendar, community stage drama reports, promotional gift items (caps, bags, jotters), posters, leaflets, stickers and fliers, IPC flipcharts and job aid cards. This paper reports on the findings of the secondary data analysis.

The objectives for the secondary data analysis were: (i) to explore the relationship between channels of LLIN-related BCC messages, their recall, and subsequent attitudes and behaviours; (ii) to make recommendations linked to these findings on strategies to intensify utilization of bed nets.

## Methods

### Study design

The surveys were undertaken in between 2009 and 2012 in ten states in Nigeria in the context of two projects: the Support to the National Malaria Programme (SuNMaP) funded by UKaid, and the NetWorks project funded by the US President’s Malaria Initiative (PMI). All surveys were planned and implemented by Malaria Consortium with fieldwork contracted out to research and marketing services (RMS). Data sets were kindly provided by Malaria Consortium.

### Study sites

The geographic location of the ten sampled states and the corresponding rainfall pattern is shown in Additional file 1. All surveys were population-representative household surveys with a cluster sampling design. Population proportionate sampling using the population at the third-level administrative unit of a ward was applied to identify the location of clusters and then villages (settlements) were selected by simple random sampling from a complete list of villages in the ward. Identification of households within the cluster was based on a comprehensive listing of eligible households on the day of the survey and households were selected using random number lists. All surveys had a single domain with no over-sampling of the urban stratum with two exceptions: Kano had implemented the campaign in two waves and each wave was defined as a separate domain; this was also the case in Cross River where, in addition, areas where a school net distribution pilot was planned were oversampled as a third domain.

A standardized questionnaire was used throughout with only minor modifications between states. The basic design was taken from the Malaria Indicator Survey (MIS) with additional modules added with respect to the registration and distribution process from the campaigns including: the hanging of nets and any difficulties encountered, retention of campaign nets and fate of previously owned nets, exposure to net related messages with source of information and the key messages recalled, as well as a section exploring attitudes, beliefs and knowledge using a set of Likert score questions, i.e., by asking respondents to choose on a scale of agreement. In November 2011 these questions were expanded for the remaining four surveys to include a broader spectrum of knowledge, attitudes and practices (KAP) statements. For the net module questions of reasons for non-use of nets were added.

### Sampling and sample size

Sample size calculations were based on the two domains of the first survey, Kano State, and aimed at detecting at least a 10 % point difference between the domains if the outcome estimate in one domain is 50 % and assuming an alpha error of 0.05, beta-error of 0.2, design effect of 1.75 and non-response rate of 5 %. This resulted in a sample of 510 households per domain (1020 total) divided into 30 clusters per domain (60 clusters total) with 17 households per cluster. This 60 × 17 design of a total of 1020 households per survey was applied to all other states with the exception of Cross River which had 75 clusters and a target sample of 1275 households as two areas were over-sampled for another project. The actual achieved samples for households, individuals and nets after data cleaning is given in Table 1 which also shows the month and year of the survey, relationship to the rainy season and timing with respect to the campaign.

### Data preparation and analysis

Data cleaning and preparation was done by Malaria Consortium analysts from data files which had been double-entered and validated against the paper forms collected in the field. An asset-based wealth index using principle component analysis was calculated and wealth quintiles created. The data sets were screened for the variables of interest and those not needed or available in all data sets were dropped. A new variable was created to identify the state so that data sets could be appended and a unique identifier be maintained for each observation, i.e., household identification (ID) plus state/survey etc. Explanatory variables for timing of the survey with respect to rains (see Table 1) and the region (north and south, see Additional file 1) were created, as was a grouped variable for the time since the campaign.

**Table 1:** Sample size and timing of surveys

State	Sample size			Timing		
	Households	Nets	Individuals	Month/year	Rainy season	Months after campaign
Sokoto	1008	1271	4468	May 2010	Dry/early	5.7
Katsina	1017	1532	4630	May 2011	Dry/early	6.5
Kano	987	1173	4642	Oct 2009	Late rains	1.9/5.4^a^
Niger	1001	1280	6270	Jun 2010	Mid-rains	6.4
Nasarawa	1015	1136	5323	Nov 2011	Late rains	10.6
Anambra	1012	1781	4546	Nov 2009	Late rains	4.1
Enugu	1020	1444	4644	Apr 2012	Dry/early	13.4
Ogun	952	745	4373	Jul 2010	Mid-rains	7.2
Lagos	1020	937	4486	Jun 2012	Mid-rains	9.0
Cross river	1254	1316	5656	Jun 2012	Mid-rains	8.2/16.5^a^
TOTAL	10,286	12,615	49,038			

^a^Two distribution waves

In order to assess the influence of intra-household net ownership on the outcomes of interest a categorical variable was created based on the relationship of number of ITN and people in the household with the following levels: (i) no ITN; (ii) fewer than one ITN/three persons; (iii) one ITN/three persons; (iv) one ITN/two persons; (v) one ITN or more/persons. The last two categories were considered as enough nets for all household members. A similar variable was created for net ownership in general with the following three categories: (i) households with no nets at all; (ii) those with any nets but no net from the campaign; and, (iii) households with at least one net from the campaigns. Exposure to any messages on nets (hanging or use) in the 6 months preceding the survey was recorded in the questionnaires together with all the sources of information. These were then grouped into five major groups, namely campaign (mobilizers, distribution point, leaflets/posters), media (radio, drama show, television, newspaper), mediators (community, political and faith leaders, town announcers), health system (health workers) and social network (friends and family).

### General approach to analysis and treatment effect model

BCC impact is generally not easy to measure as the effects are complex and not caused by a single event of exposure to a message, but rather by repeated exposures from multiple sources modified by various internal and external factors. Therefore, three steps were used to define BCC exposure-dependent outcomes as a composite measure of the BCC effects, which could then directly be linked to net hanging and use in univariable and multivariable analysis: (i) exposure to messages about net hanging and use was explored with respect to coverage (any exposure) and intensity (number of information sources)^1^; (ii) the link between recall of any content and that of specific messages was explored to establish whether there is a dose–response relationship between exposure and recall; and, (iii) an association between message recall and specific KAP outcomes (confidence in taking action to protect the family with nets, reported discussing of net use within the family^2^ and expressed intention to use the nets every night^3^) relevant to hanging and using of nets was explored. For the Likert score questions response options were recoded to read 2 for “definitely could”, 1 for “probably could”, −1 for “probably could not”, −2 for “definitely could not”. Similarly, the responses were recoded to read 2 for “strongly agree”, 1 for “somewhat agree”, −1 for “somewhat disagree”, and −2 for “strongly disagree”. Attittude, knowledge and perception scores were then calculated as the mean score of all questions in that group after the value of any negatively phrased questions had been inversed. With this approach a mean of 0 represents a neutral response, below zero a negative response and above zero a positive response. The scores were then grouped into four categories: <0 as poor response, 0–0.99, 1.0–1.49 and 1.50 or higher as excellent response. Further information on the analysis approach can be found in Additional file 2.

To quantify the treatment effects from BCC, i.e., the impact of BCC exposure on ITN use, a treatment effect model was used with an inverse-probability weight regression adjustment (IPWRA in Stata). Treatment effect models use predicted exposure to an intervention (here BCC) as a variable in a simultaneous equation that predicts the outcome behaviour of interest [20], thereby estimating the counterfactuals (what would have happened if an exposed person had not been exposed, etc.) in a quasi-experimental approach from within observational data. For these models fulfilment of underlying assumptions was tested before results were used.

### Uni- and multi-variable analysis

Statistical analysis was done with STATA 13.1 (StataCorp, Texas, USA). All cross tabulations and testing of means in the initial univariable analyses were done applying the sampling weights from the original surveys and adjusting 95 % confidence intervals (CI) for the design effect of the cluster sampling design by using the appropriate survey commands (SVY). In addition the survey was defined as second level of cluster in the analysis. All models also applied the survey analysis settings.

From the univariable analysis, potential confounders and explanatory variables were identified and then the key effects explored in multivariable linear or logistic regression models. In general, terms in the models were kept if they had a marginal significance with *p* < 0.2. However, due to the characteristics of the data set comprising ten surveys from different years and states within Nigeria, the variables year of survey, season, time since campaign, and region were maintained in all models irrespective of significance. These variables were used instead of the state itself in order to allow the separate analysis of specific aspects, such as seasonality, which would have otherwise been absorbed in the state variable thereby confounding the results. Fit of the models was assessed by the coefficient of determination (R-squared) and by likelihood-ratio tests.

## Results

### Exposure to and recall of messages on net hanging and use

#### Message recall

**Fig. 1:**
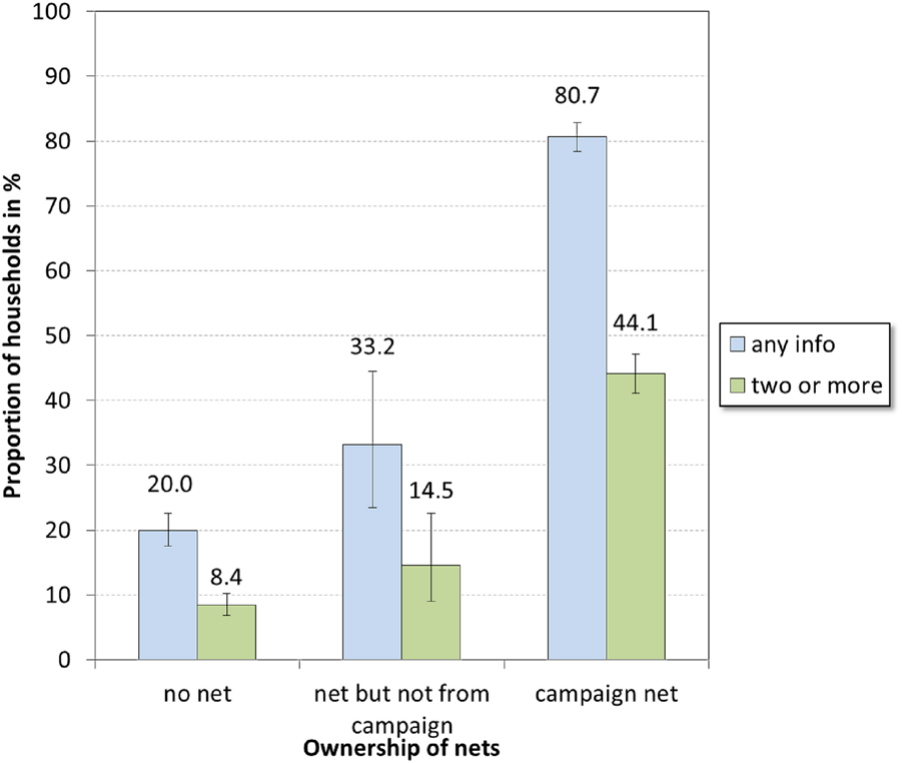
Exposure and recall of messages in relation to net ownership

More than half of the respondents (58.4 %; 95 % CI 56.0, 60.7) reported having heard any message about net hanging and use during or after the campaign, ranging from 45.9 % in Kano to 70.5 % in Enugu. The rate of recall increased with time since the campaign from 37.9 % in the first 3 months, to 56.8 % 3–9 months afterwards, and to 63.8 % after 9–16 months afterwards. Recall of messages was seen to be dependent on the ownership of nets (Fig. 1); in households with no nets, recall of any message was 20.0 %, in households with any nets (but not nets from the campaign) it was slightly higher at 33.2 %, and in households with campaign nets, it was four times higher (80.7 %) than households with no nets.

#### Determinants of information exposure

The different sources of information were analysed by all households and by those that were able to recall any information. Media (mainly radio messages or songs) were the most commonly cited information sources in both groups (21.5 and 36.8 %, respectively), followed by information channels directly linked to the campaign (campaign leaflets and information from the mobilization team) with 19.7 and 33.7 %. This was followed by health systems (through a health worker) with 18.6 and 31.9 %, and mediators (such as local or political leaders, town announcers) with 15.7 and 26.8 %. Social networks (family or friends) were the least cited source with 14.8 and 25.4 %. A detailed breakdown by sub-category is shown in Additional file 3.

The determinants of information exposure were explored in a multivariable logistic regression model (Table 2) and stratified by two outcomes: households that had any exposure to BCC messages, and households that could recall two or more messages. In addition to net ownership and time since distribution, which showed a significant association with information exposure to both outcomes, only two other factors showed a strong association among households that had received any exposure: small households without children were much less likely to have received any message on net hanging and use (odds ratio (OR) 0.69, *p* < 0.001), whilst owning a radio (independent of socio-economic status) was positively associated (OR 1.37, *p* = 0.022). Among households that could recall two or more messages, only small households without children were significantly less likely to have received any message (OR 0.69, *p* < 0.001).

**Table 2:** Multi-variable logistic regression of exposure to information and message recall

Explanatory variable^a^	Outcome 1: any exposure			Outcome 2: recall two or more messages		
	OR	95 % CI	*p* value	OR	95 % CI	*p* value
Net ownership						
No net	1.00	–	–	1.00	–	–
Net but not from campaign	2.20	1.4, 3.5	0.001	2.12	1.2, 3.8	0.012
Campaign net	18.02	14.5, 22.4	<0.0001	9.62	7.6, 12.1	<0.0001
Time since campaign						
0–3 months	1.00	–	–	1.00	–	–
3–6 months	2.54	1.2, 5.4	0.015	4.87	2.0, 11.7	<0.0001
6–9 months	3.82	1.4, 10.7	0.11	6.89	2.3, 20.4	0.001
9–16 months	4.73	1.8, 12.5	0.002	9.05	3.2, 26.0	<0.0001
Wealth quintiles						
Poorest	1.00	–	–	1.00	–	–
Second	0.90	0.7, 1.2	0.42	0.87	0.7, 1.1	0.17
Third	0.83	0.6, 1.1	0.23	0.81	0.6, 1.0	0.10
Fourth	1.21	0.9, 1.6	0.20	1.12	0.9, 1.4	0.37
Wealthiest	1.13	0.8, 1.5	0.04	1.10	0.8, 1.4	0.47
Education of head						
Non-literate	1.00	–	–	1.00	–	–
Primary	1.02	0.8, 1.3	0.88	1.00	0.8, 1.2	0.98
Secondary	1.23	0.9, 1.6	0.10	1.34	0.9, 1.4	0.27
Tertiary and higher	1.18	0.9, 1.6	0.27	1.11	0.8, 1.5	0.48
Age of head in years						
19–29	1.00	–	–	1.00	–	–
30–59	1.15	0.8, 1.5	0.30	1.19	0.9, 1.6	0.23
60+	1.29	0.9, 1.8	0.12	1.29	0.9, 1.7	0.092
HH with 3 or less people and no children	0.69	0.6, 0.8	<0.0001	0.69	0.6, 0.8	<0.0001
HH head is female	1.20	0.9, 1.5	0.075	1.14	0.9, 1.4	0.18
HH has radio	1.37	1.0, 1.8	0.022	1.14	0.9, 1.4	0.18
Urban versus rural	0.98	0.7, 1.3	0.85	0.93	0.7, 1.1	0.44
North versus south	0.76	0.5, 1.2	0.20	1.34	0.9, 2.0	0.14

^a^The variables year and season of survey were included in models to adjust for timing

#### Complementarity of information exposure and sources

Considering that the overall result of information exposure is the sum of the effects of each of the five communication channels, each of these channels was examined in a separate regression model to determine the complementarity of their profiles (whether specific channels are better suited to reach particular sections of the target population) and detailed results are shown in Table 3. Recall of campaign BCC was seen to increase over time after the campaign (*p* < 0.001), slightly favouring wealthier households (OR 1.3, *p* = 0.023) and was strongly associated with the first year of campaigns (OR 3.3, *p* = 0.004). Individuals who recalled campaign BCC were significantly more likely to own a campaign net (OR 32.3, *p* < 0.001). Interestingly, recall of campaign BCC had significantly lower odds in the north (OR 0.49, *p* < 0.001). The health system channel was strongly associated with the campaign: those exposed to this information channel had significantly higher odds of owning a campaign net (OR 6.2, *p* < 0.001) and had a similarly strong increasing trend after the campaign (*p* < 0.001). Households with a better educated head of household were most likely to recall messages from this source (OR 1.2, *p* = 0.003), whilst small households with no young children (OR 0.67, *p* < 0.001) and households with older heads of household (OR 0.86, *p* = 0.007) were less likely to pick up messages from the health system. Communication through mediators, also associated with the campaign, showed a strong increase with time since the campaign (OR 3.5, *p* = 0.007) but otherwise reached exactly those who were less favoured by the health system, namely older household heads (OR 1.5, *p* = 0.006) and those with lower education (OR 0.76, *p* = 0.011). In addition, this channel was more associated with the northern campaigns (OR 3.3, *p* < 0.001) and appeared to be more emphasized in the early campaigns (OR 2.4, *p* = 0.076). Media-related BCC was positively associated with radio ownership (OR 2.7, *p* < 0.001). Media-related BCC also favoured wealthier (OR 1.3, *p* = 0.001) and better-educated families (OR 1.3, *p* = 0.008) and was marginally favouring disadvantaged small families without young children (OR 0.84, *p* = 0.069). Finally, BCC through social networks was linked to urban (OR 1.7, *p* < 0.001), wealthier (OR 1.3, *p* = 0.033) and younger households (30–59 years OR 0.75; 60+ years OR 0.85, *p* = 0.069) with young children (OR 0.66, *p* < 0.001). Communication through this channel appears to be more emphasized in the late campaigns (OR 0.26, *p* < 0.001).

**Table 3:** Determinants of exposure to different information channels; results from multi-variable logistic regression models

Variable	Information channel				
	Campaign	Health worker	Mediators	Media	Social networks
Has campaign net versus non-campaign nets	OR 32.3 *p* < 0.001	OR 6.2 *p* < 0.001	OR 3.5 *p* = 0.007	OR 2.4 *p* = 0.005	OR 1.0 *p* = 0.99
Time since campaign^a^	Increasing *p* < 0.001	Increasing *p* < 0.001	Increasing *p* < 0.001	Neutral *p* = 0.20	Decreasing *p* < 0.001
Wealth quintiles 4/5 versus 1–3	OR 1.3 *p* = 0.023	OR 1.0 *p* = 0.93	OR 0.91 *p* = 0.36	OR 1.3 *p* < 0.001	OR 1.3 *p* = 0.033
Education of head Secondary or higher	OR 1.1 *p* = 0.50	OR 1.2 *p* = 0.033	OR 0.76 *p* = 0.011	OR 1.3 *p* = 0.008	OR 0.92 *p* = 0.50
Age of head of HH					
15–29 30–59 60+	– OR 1.2 OR 1.1 *p* = 0.53	– OR 1.1 OR 0.86 *p* = 0.077	– OR 1.1 OR 1.5 *p* = 0.006	– OR 1.2 OR 1.2 *p* = 0.43	– OR 0.75 OR 0.89 *p* = 0.069
Gender of head Female versus male	OR 1.0 *p* = 0.77	OR 0.87 *p* = 0.20	OR 1.0 *p* = 0.74	OR 1.2 *p* = 0.10	OR 1.1 *p* = 0.43
Family size Less than 3^b^ versus more	OR 0.91 *p* = 0.39	OR 0.67 *p* < 0.001	OR 0.85 *p* = 0.11	OR 0.84 *p* = 0.069	OR 0.66 *p* = 0.001
Residence Urban versus rural	OR 0.88 *p* = 0.31	OR 1.2 *p* = 0.21	OR 0.82 *p* = 0.15	OR 0.85 *p* = 0.20	OR 1.7 *p* = 0.001
Region North versus South	OR 0.49 *p* < 0.001	OR 0.74 *p* = 0.15	OR 3.3 *p* < 0.001	OR 1.3 *p* = 0.17	OR 0.95 *p* = 0.77
Radio ownership versus none	OR 0.91 *p* = 0.43	OR 1.0 *p* = 0.66	OR 0.89 *p* = 31	OR 2.7 *p* < 0.001	OR 1.1 *p* = 0.52
Year 2009 versus 2010–2012^c^	OR 3.3 *p* = 0.004	OR 0.83 *p* = 0.66	OR 2.4 *p* = 0.076	OR 1.2 *p* = 0.62	OR 0.26 *p* < 0.001

*HH* head of household

^a^See also Fig. 3

^b^Without any children under 5

^c^Early campaigns = Kano and Anambra

### Relationship between information sources and messages recalled

**Fig. 2:**
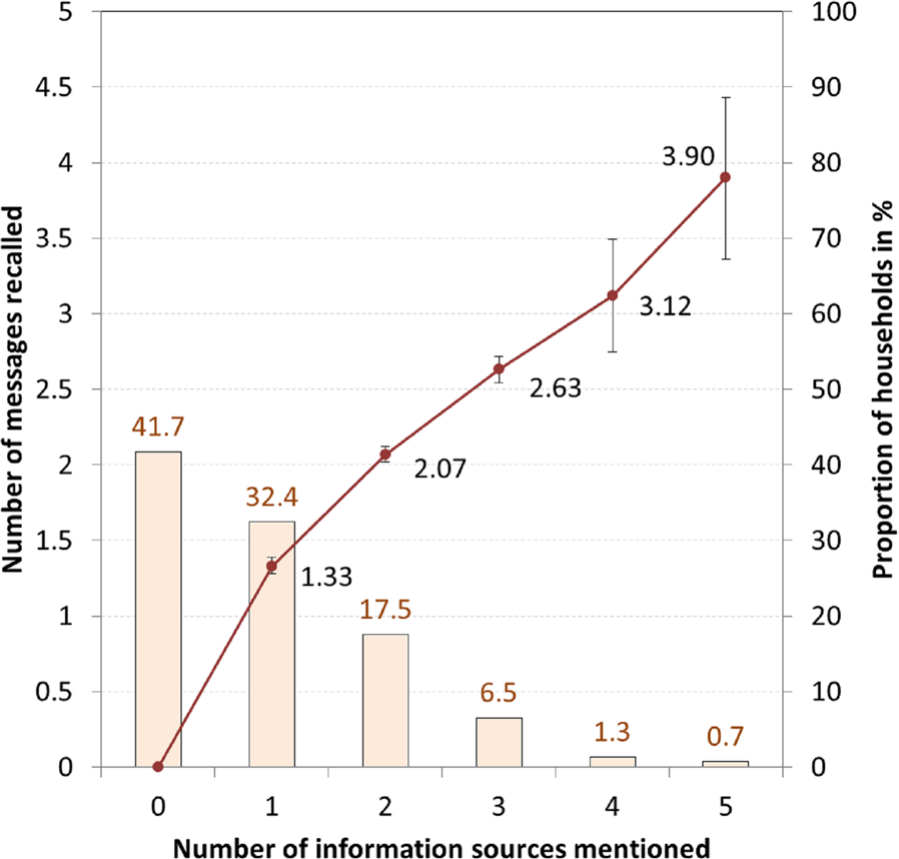
Multiple channel information and relationship to number of messages recalled. *Bars* proportion of households recalling messages; *line* mean number of messages recalled

Figure 2 depicts a strong relationship between the number of information sources mentioned by the respondent and the number of messages recalled, linearly increasing from 1.3 if only one source was mentioned to 3.9 messages recalled for five or more sources. In a linear regression this relationship was statistically significant (*p* < 0.0001) with a high coefficient of determination (R-squared 0.70) meaning that 70 % of the variation of messages recalled in the data set can be explained by the number of information sources alone. However, only close to half (44.4 %) of those with any information and 25.9 % of all respondents could recall more than one source of information and 14.4 and 8.4 %, respectively, three or more information sources. Subsequently, only 30.9 % (95 % CI 28.8, 33.1) of respondents recalled two or more messages, and this proportion was somewhat higher among those who had received any nets from the campaign reaching 44 %.

Finally, the relationship between specific messages and communication channels was explored using the variables previously identified as determinates of exposure and results are presented in Table 4. They show that the message on net hanging was mainly carried by the campaign and health workers, but very little by the media or social networks while on the other hand “use net every day” was strongly associated with media and “nets prevent malaria” was most closely linked to communication through family and friends.

**Table 4:** Adjusted odds ratios of recall of specific messages by information channel

Message	Information channel—adjusted OR (95 % CI)				
	Campaign	Health worker	Mediators	Media	Social networks
Use net	3.4 (2.7, 4.3)	2.2 (1.7, 2.7)	3.5 (2.8, 4.6)	3.3 (2.6, 4.1)	2.6 (2.0, 3.4)
Sleep under net every night	3.3 (2.6, 4.1)	3.9 (3.1, 4.8)	3.8 (2.8, 5.3)	5.3 (4.2, 6.8)	2.4 (1.8, 3.0)
Hang net	4.2 (3.3, 5.2)	3.5 (2.8, 4.2)	3.0 (2.3, 3.7)	1.9 (1.6, 2.4)	1.7 (1.3, 2.1)
Nets prevent malaria	3.0 (2.4, 3.7)	3.7 (2.9, 4.6)	3.7 (2.9, 4.7)	2.8 (2.2, 3.4)	6.2 (4.8, 7.9)
Value net	3.4 (2.5, 4.4)	2.6 (2.0, 3.5)	3.0 (2.3, 3.8)	3.4 (2.6, 4.5)	2.5 (1.9, 3.4)

### BCC outcome measures

BCC outcomes were defined as the effects of message exposure and recall on knowledge, attitudes, perception as well as intentions to use nets. Measurements of KAP and belief were measured using the Likert score questions (see Additional file 4). Ability to take action to protect the family using ITN, discussing net use within the family and intended use of nets every night were used to measure attitudes, whilst responses to particular statements were used to measure knowledge and perceptions/beliefs (see Additional file 4). These factors were then entered into separate multivariable regression models to assess the effect of explanatory variables.

### Attitudes towards net use

The ability to take action to protect the family using ITN was the major measurement of attitudes towards net use and data were available for all ten surveys. When entered into a multivariable regression model, being confident to take action to protect the family with ITNs was positively linked to the number of messages recalled (details presented in Additional file 5), indicating that the mean action score increased significantly with each additional message recalled, with the strongest effect for recall of four (coefficient: 0.25, *p* < 0.001) or five (coefficient: 0.3, *p* = 0.001) messages. Confidence to take action was significantly linked to the rainy season (mid-rainy season coefficient: 0.48, *p* < 0.001), owning any net (coefficient: 0.17, *p* < 0.001) and heads of household having secondary (coefficient: 0.16, *p* < 0.001) or tertiary (coefficient: 0.18, *p* < 0.001) education. The action score was highest 6–9 months (coefficient: 0.36, *p* < 0.001) and nine to 16 months (coefficient: 0.18, *p* < 0.001) following the campaign. Two factors decreased a household’s confidence to take action: the age of the head of household (aged 30–59 coefficient: −0.06, *p* < 0.001; aged 60+ coefficient −0.14, *p* = 0.002), and small families without children (coefficient −0.09, *p* = 0.001). Participation in the campaign, urban versus rural residence and gender of the head of the family had no statistically significant influence.

**Fig. 3:**
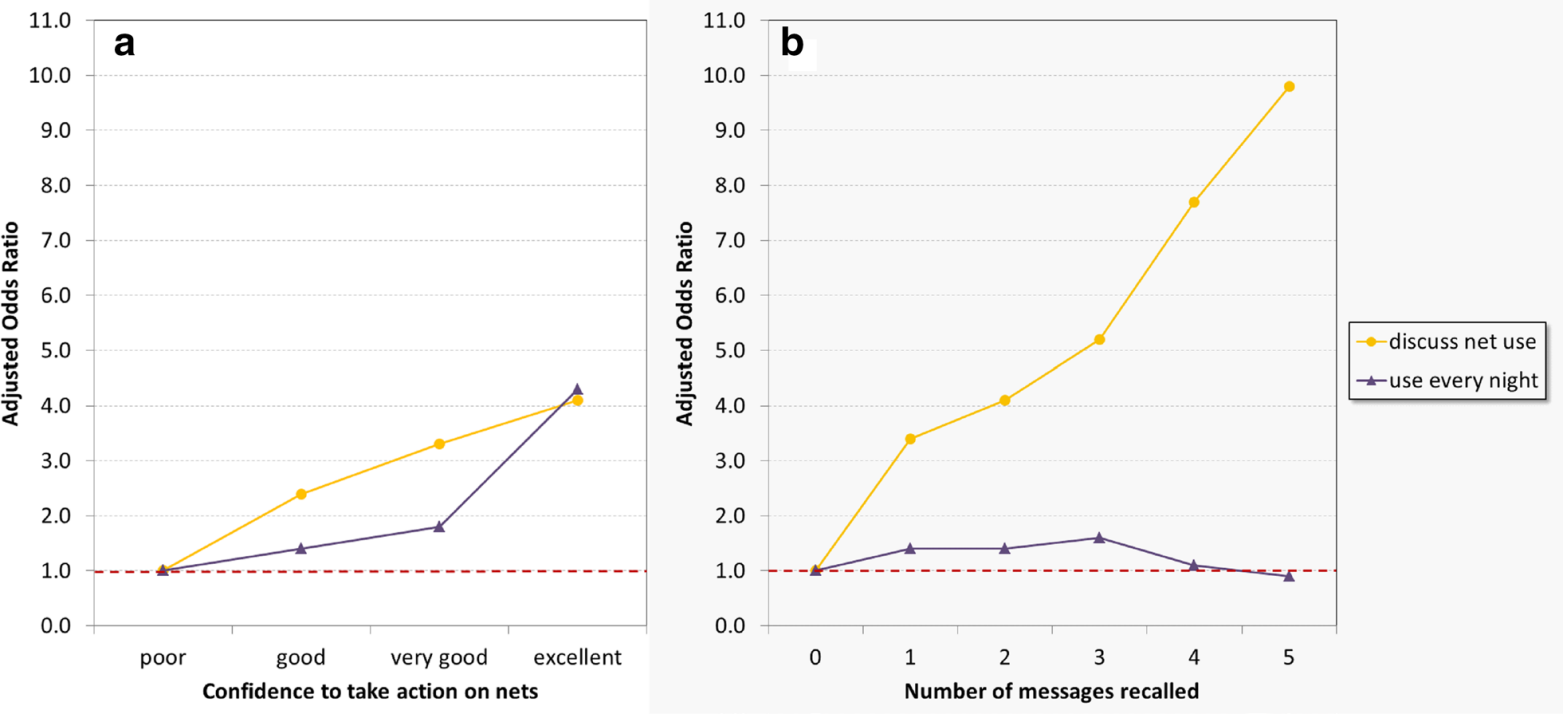
Effect of BCC message recall (**a**) and confidence to take action on nets (**b**) on BCC outcomes

Discussing net use within the family and intended use of nets every night were entered into a multivariable linear regression model (details shown in Additional file 5). Discussing net use with family showed a dose–response link with BCC messages and had the strongest single explanatory component (*p* < 0.001), responsible for 16 % of the variation in the response or 56 % of the overall coefficient of determination of the model (R-squared 0.29). On the other hand, intention to use net every night was only slightly influenced by BCC message recall without a dose–response relationship explaining merely 15 % of the overall variation explained by the model (R-squared 0.15). In contrast, both variables were linked to the confidence to take action although, again, the intention to use every night showed less of a dose–response (Fig. 3). Whilst for discussion, the second-most important determinant was owning any net and specifically a campaign net (OR 7.33, *p* < 0.001), it was the rainy season and particularly the peak of the rains for use every night (OR 4.79, *p* < 0.001).

Regarding regional differences, the north showed significantly better results for both variables meaning that for all three KAP measures for attitudes in this analysis [confidence to take action (OR 0.40, *p* < 0.001), discussing net use in family (OR 1.65, *p* < 0.001) and intention to use every night (OR 1.45, *p* < 0.001)], the ‘net culture’ was better in the north—all other aspects being equal, i.e., adjusting for potential confounders such as wealth, seasonality, education, and level of exposure to messages. The models also identified some variables with a moderate negative effect, i.e., they reduced the likelihood that net use was discussed or an intention to use every night was expressed. These factors were non-literate heads of households for both variables [discussing net use in family (OR 0.80, *p* = 0.011) and intention to use every night (OR 0.70, *p* < 0.001)], young heads of households (OR 0.75, *p* = 0.003) and small families without young children (OR 0.73, *p* = 0.001) for discussion and urban residence (OR 0.69, *p* = 0.008) for use every night.

### Knowledge and perceptions/beliefs about net use

Statements on knowledge about ITN and perceptions and beliefs about their use were available for only 4309 (42 %) households from four states (Nasarawa, Enugu, Lagos, Cross River). In a multivariable linear regression model the number of messages recalled was the strongest predictor of knowledge (*p* < 0.001); there was a clear dose–response relationship, showing knowledge increased with each additional message remembered, resulting in a proportion of 25 % of households with excellent knowledge if four of more messages were recalled. There were no major misconceptions to perceptions and beliefs.

### Impact of BCC outcomes on net use

#### Net use by individuals

Among the 49,033 individuals registered in the surveys 47,703 (97.5 %) stayed in the house the previous night and represent the *de*-*facto* population. Among these 44.6 % (95 % CI 42.4, 46.8) had access to an ITN, assuming that each net was used by two people. Of the *de*-*facto* population, 29.3 % (27.2, 31.2) had used any net and 28.1 % (26.2, 30.0) had used an ITN the previous night. This means that overall 62.9 % (59.6, 66.8) of those with access used an ITN the previous night or 37.1 % (33.2, 40.4) did not use an ITN. The latter is termed the ‘use gap’.

Multivariable logistic regression (Table 5) among households with any nets verified that the strongest positive influence on net use was the intra-household net supply, with dose–response relationship, i.e. increasing use with increasing number of nets in houseold, and a three-fold increase in the likelihood of a person having used a net last night if there is at least one net for every two people (OR 3.38, *p* < 0.001) compared to less than one net for every three people (OR 2.23, *p* < 0.001). The model also confirms the strong impact of increasing confidence to take action (*p* < 0.001), expressing the intention to use nets every night (OR 2.32, *p* < 0.001), and discussing net use (OR 1.56, *p* < 0.001). Similarly, net use was 2.5 times more likely if the household had not reported any difficulties in hanging of nets (OR 0.40, *p* < 0.001). Adjusting for all other factors, net use was significantly higher during the mid-rains or peak of rainy season (OR 1.53, *p* = 0.005) while there was still an increase in the late rains, i.e. at the end of the rainy season (OR 1.36, *p* = 0.18), but this was only marginally significant. At the same time, net use was stronger in the north (OR 1.63, *p* = 0.003) and the change of use with seasons was significantly different in the north than in the south (*p* < 0.0001 for interaction term in model) suggesting a significant seasonal variation in the north and no or a weak one in the south.

**Table 5:** Multivariable logistic regression of net use if households own any nets

Explanatory variable^a^	Outcome: net use last night		
	OR	95 % CI	*p* value
Confidence to take action on nets			
Poor	1.00	–	–
Good	1.45	1.1, 1.9	0.006
Very good	1.76	1.3, 2.3	<0.001
Excellent	1.93	1.5, 2.5	<0.001
Discussing net use			
Discuss versus no discussion	1.56	1.3, 1.9	<0.001
Intention to use net			
Use every night versus less frequent	2.32	2.0, 2.7	<0.001
Difficulties in hanging net			
Did report versus not	0.40	0.3, 0.6	<0.001
Season			
Dry	1.00	–	–
Mid-rain	1.53	1.1, 2.1	0.005
Late rain	1.36	0.9, 2.1	0.18
Supply with nets			
Less than 1/3 persons	1.00	–	–
1/3 Persons	2.23	1.9, 2.7	<0.001
1/2 Persons or better	3.38	2.9, 4.0	<0.001
Region			
North versus south	1.63	1.2, 2.3	0.003
Residence			
Urban versus rural	0.82	0.7, 1.0	0.040
Age and gender group			
Males 15–49	1.00	–	–
Children under 5 (M and F)	2.29	1.9, 2.7	<0.001
Children 5–14 (M and F)	1.45	1.3, 1.7	<0.001
Women 15–49 not pregnant	1.70	1.5, 1.9	<0.001
Women 15–49 pregnant	2.13	1.7, 2.7	<0.001
Age 50+ (M and F)	1.31	1.1, 1.6	0.001
Relationship			
Is head of household versus other members	1.57	1.4, 1.8	<0.001

^a^The variables time since campaign and year were included in models to adjust for differences in surveys

The relationship between age and gender and net use was explored by constructing different age-gender groups, and using men age 15–49 years of age as the reference point. The results show that this group had the lowest net use, followed by people aged 50 or more (OR 1.31, *p* = 0.001) and older children five to 14 years (OR 1.45, *p* < 0.001). The highest use was seen in children under five (OR 2.29, *p* < 0.001) and pregnant women (OR 2.13, *p* < 0.001), followed by women of reproductive age but not currently pregnant (OR 2.13, *p* < 0.001). Heads of household, independent of age or gender, also had a significantly increased use rate (OR 1.57, *p* < 0.001), which was above that of older children, but lower than women in reproductive age. Finally, there was a significantly lower use in urban areas (OR 0.82, *p* = 0.04) but no independent effect of wealth quintiles, education or gender of the head of household.

#### Estimation of treatment effects on net hanging and use

Treatment effects were modelled for the main outcome of population net use with the three major BCC outcomes as the treatment variables. The treatment model included those variables that had been shown to influence the BCC outcome, namely: number of messages recalled, participation in campaign, exposure to specific media channels and residence as well as year, time since campaign, and region of the survey in order to adjust for the structure of the data. The outcome model included those variables that were strongly associated with net use namely intra-household net supply, season, region, age and gender groups, and residence, but also the other BCC outcomes in order to adjust for confounding from these factors.

Results are shown in Table 6 and indicate that treatment effects from the models were generally smaller than indicated by the crude, univariable tabulation suggesting that some of the differences were not caused by the BCC outcomes but rather by either confounding variables or colinearity among the variables included in the model. Nonetheless, all the outcomes showed a significant increase in net use, which was strongest for the confidence to take action which increased by 4.4 %-points at each level with an overall effect of 17 %-points increase of net use comparing poor and excellent confidence levels. Intention to use every night increased net use by 15 %-points and discussing net use in the family by 8 % points. All these effects were statistically significant (*p* < 0.001). However, there was no indication that these effects were additive as an outcome variable that included the combination of a high confidence with intention to use every night did not show a higher treatment effect that any of the two alone.

**Table 6:** Treatment effects of BCC outcomes on net use in households with any net

BCC outcomes	Outcome: population net use			
	Univariable		Treatment effects model	
	Estimate (%)	95 % CI	Adjusted estimate (%)	95 % CI
Confidence to take action on nets				
Poor	22.9	18.6, 27.9	27.4	25.1, 29.7
Good	32.9	29.8, 36.2	36.5	35.2, 37.8
Very good	41.7	38.8, 44.7	42.5	41.5, 43.6
Excellent	49.7	47.4, 52.0	44.9	44.1, 45.6
Treatment effect as difference in use (poor vs. excellent)	26.8		17.4	15.0, 19.0
Intention to use net				
Use less than every night	32.2	29.8, 34.6	35.1	34.3, 35.9
Use every night	54.5	52.3, 56.6	50.5	49.5, 51.4
Treatment effect as difference in use	22.3		15.4	14.2, 16.6
Discussing net use				
No discussion	30.6	26.6, 34.9	34.7	33.1, 36.3
Discuss	44.7	42.7, 44.6	43.1	43.0, 44.2
Treatment effect as difference in use	14.1		8.4	6.7, 10.1

## Discussion

This analysis of ten post-campaign surveys from Nigeria attempted to identify the impact of BCC message exposure on net hanging and use and describe the effects in as much detail as possible in order to inform the design and implementation of the second wave of LLIN mass campaigns in Nigeria. The main findings can be summarized as follows.

### Message exposure and recall

Exposure to net-related messages in the previous 6 months was overall quite high with 58 % of household respondents stating that they had heard or seen any messages. Interestingly, results showed that different communication channels differed significantly not only in whom they reached, but also in what messages they prioritized.

Exposure to the campaign channel was seen to increase over time, which could be an effect of people reading leaflets some time after the campaign or being reminded of the exposure by seeing posters in the months following the distribution. Recall of campaign BCC slightly favoured wealthier households and was significantly less in the north, which is most likely due to the fact that two of the five northern campaigns were integrated with polio-immunization where BCC focused more on the vaccination. Interestingly, campaign-related communication was strongly associated with the first year of campaigns, i.e., Kano and Anambra, suggesting that special effort was put into these from all stakeholders that may not have been present in the later campaigns. The campaign BCC (leaflet, poster and teams) worked best to convey the “hang your net” message and was most intensive in the first year of campaigns. In the late 2000s it was hypothesized that lack of ITN use was attributed to difficulties in hanging ITNs and poor knowledge regarding their use [25]. Resultantly, WHO now recommends including hang-up campaigns by community volunteers as part of LLIN distribution campaigns [8]. However, the impact of post-campaign visits on ITN use has been debated, with published literature from various countries in sub-Saharan Africa finding no significant impact in use following visits [17, 26–28]. However, this study has indicated that the campaign was critical in the study population being exposed to net-related information.

Messages from the campaign were reinforced by health workers during subsequent health facility visits, resulting in a still very strong association in health worker communication with the campaign and similarly strong increasing trend after the campaign. It clearly favoured households with better educated head of household—possibly because this group is more likely to seek treatment—and disadvantaged small households without any young children who either are also less likely to frequent the facility or were not seen as a target for net messages by the health workers. There was a marginally lower pick up of messages from health workers by older heads of household. Communication through mediators (local and political leaders, town announcers) also reinforced the net messages following the campaign and showed a strong increase with time since the campaign, but otherwise reached exactly those who were less favoured by the health system, namely older household heads and those with lower education. This channel was more associated with the northern campaigns and appeared to be more emphasized in the early campaigns (Kano and Anambra).

Media communication mainly comprised radio messages, songs and community drama shows and was independent of the time after the campaign. It was, not surprisingly, the only channel that was associated with radio ownership. Media-related BCC favoured wealthier and better-educated families and marginally favoured female headed households (possibly due to radio listening during the day) and disadvantaged small families without young children. Mass media (mainly radio) was best in communicating the message “sleep under a net every night”. A previous study in Nigeria also found that respondents who listened to radio were approximately 1.6 times more likely to use an ITN (OR 1.56; 95 % CI 1.07–2.28; *p* = 0.020) [24], and findings from the BCC/IEC report in Nigeria found that several respondents mentioned the use of radio-based BCC strategies (jingles and drama series) in northern Nigeria.

Although social networks through family and friends are informal and not directly implemented as an external BCC activity, they not only had a significant role in providing households with net-related information, but also had a different reach compared to the other channels in that they were totally independent of net ownership, i.e., it happened in a similar way whether households owned campaign nets or not. It showed a peak in the three to 6 months after the campaign, declining thereafter and was linked to urban and younger households with young children. Communication through this channel also appeared to have picked up some time after the start of the campaigns, which would suggest that it needed some momentum from the other channels to get started. In Bangladesh, interpersonal communication with community health workers, relatives, neighbours and friends was found to be an important source of acquiring malaria information [29], and results from a quasi-experimental evaluation in Zambia concluded that interpersonal communication activities were likely to have contributed to an observed increase in ITN use in Zambia [30]. Communication through family and friends (social networks) had the strongest effect for the message “nets prevent malaria” and was unique in that it worked equally well for households that had not participated in the campaign.

Whilst the campaign-related BCC activities were critical to reach high exposure levels, no single communication channel alone could reach more than one-fourth to one-third of the households, providing strong evidence that a multi-channel, multi-media approach is needed to reach these kinds of BCC exposure levels. The NMEP and its partners would be wise to continue to implement a multi-channel, multi-media approach to BCC for the support of a strong net culture that focuses on clear messages and is not limited to the distribution campaign, but extends through other channels in order to re-enforce the effects of BCC. In addition to the routine channels of campaign activities, health staff and media (radio, drama, etc.) other mechanisms, especially local leaders and social networks (family or friends) should be utilized or stimulated as these are able to reach groups not easily reached by the other channels.

Some messages needed less intensive communication than others with “use net every night” and “hang your net” reaching good recall after exposure from only one or two information sources, while “value your net” appeared to have to be presented by at least three or four sources before similar recall levels were reached. There was also very strong evidence for a dose–response relationship between the number of information sources and the number of messages recalled, i.e., the more different channels and media people were exposed to, the more messages they could recall. Intensity of communication (i.e., frequency of exposure, levels of exposure, familiarity with messages transmitted through specific communications campaigns), has previously been suggested as an important factor which can affect ITN use [14]. The determinants of recalling two or more messages were very similar to those of being exposed to any information, but with a stronger increase with time since campaign which fits well with the interpretation that different communication channels reinforced messages during that period leading to increasing multiple exposures and hence increasing recall of more than one message.

### Net culture in northern and southern states

Another factor that positively influenced BCC outcomes was seasonality; during the rainy season, particularly at the peak phase of rains, there was a consistently better outcome in the northern states, suggesting a stronger or faster net culture development at the same level of exposure and recall. The relationship between rains and net use in households with any nets varied between the north and the south; during the rains, net use was higher in the north compared to the south while in the dry season the picture was reversed. This could explain the differences that were found between the 2013 Demographic and Health Survey (DHS), with higher use among those with access in the south, and the 2010 MIS, which had described significantly better usage in the north. While the MIS was undertaken during the late rains, the DHS was done early in the year during the dry season. Adjusting for the seasonality of use, the data provide evidence that net use is overall better in the north, and in keeping with the finding of a consistently higher level of net culture in the north, hanging was significantly better there compared to the south and there was also a reduced likelihood of hanging in urban compared to rural areas.

### Individual ITN use

A critical question is: who is using the nets and are there preferential age or gender groups which have to be addressed by specific or additional BCC strategies or messages? This was assessed by age- and gender-specific use rates separately for households that have at least one net for every two people. Pregnant women and young children were most likely to use a net, indicating that messages on preferential use of young children, pregnant women and young mothers are being largely followed. Non-pregnant women of reproductive age were the second-most likely group to use an ITN. Older children and men 15–49 were least likely to have used a net the previous night. Low use of ITN among children aged five to 15 years has been attributed to available ITNs being used for younger siblings and/or pregnant women/mothers, in line with IEC/BCC messages targeted to at-risk communities [31]. Low ITN use by males, especially in early adulthood, has been documented in Nigeria, with females significantly more likely to use ITNs compared to males [32].

It has been suggested that in countries where there is only a small proportion of children not using ITNs, IEC/BCC efforts should shift from promoting net use among these target groups to focussing efforts on improving one of the other categories of non-use, such as increasing ownership or hanging nets [10], or promoting use among older children and young male adults where use is reportedly low. Among communities who already give priority to vulnerable members, emphasizing this practice may be better as periodic re-inforcement rather than placing major emphasis on it in an ITN programme strategy [33]. Nonetheless, given the vulnerability of pregnant women and young children and their elevated risk of developing severe malaria, ensuring that their use remains high is essential. Eisele et al.’s review of net use among children and pregnant women in 15 sub-Saharan African countries found that many children and pregnant women are still not sleeping under ITNs from ITN-owning households, that in 60 % of countries analysed less than 50 % of children were using ITNs, while in 28.6 % of countries, less than 50 % of pregnant women were using them [13].

### Access to ITNs

It is seen that preferential net use by different groups is primarily due to the lack of nets and preference being given to children and women is very much the same for all age and sexes once there are enough nets for all household members. Among the study population, there was a three-fold increase in the likelihood of a person having used a net the previous night if there was at least one net for every two people compared to less than one net for every three people. ITN to occupant ratio has been seen as a the main drivers for ITN use, wherein children in ITN-owning households with better intra-household access to an ITN were significantly more likely to have slept under an ITN the night before [13]. In Zambia, intra-household access to an ITN was associated with higher ITN use among children [14]. Access to ITNs was highlighted as a strong factor that determined ITN use in two states in northern Nigeria; in Edo, unavailability of ITNs was the main factor affecting use [34] and in Kwara, 20 % of women cited non-availability of ITNs for purchase as a reason for not having used an ITN [35].

There was the significantly reduced likelihood of net hanging, and even more so a household having all their nets hanging, if there was an excess of nets, i.e., more than one net for every two people. In fact, during the rains households with a positive action score had all their nets hanging in 83 % of cases if they had fewer than enough nets for all members and only 45 % if they had excess nets. This suggests that the household supply situation with nets needs to be taken into account when net use and the potential impact of BCC is assessed in order not to confuse none-use due to surplus nets with unwillingness to use.

In the overall sample, 37 % of household members who had access to a net had not used it the previous night. Kilian et al. found that the use gap differed between northern and southern geopolitical zones in Nigeria, with a use gap of 11 and 36 %, respectively [17]. In this study, the use gap was strongly influenced by the BCC-related outcomes reducing the use gap from 66 % if a household had poor confidence to take action to only 29 % gap if it was excellent. Similarly, intention to use the nets every night reduced the use gap from 52 to 19 %. This indicates that BCC does have a positive influence on ITN use, if there are sufficient ITNs available for household members.

Analysis of knowledge and perceptions about ITNs and their use in four of the ten states also revealed that there is some misconception about the possible harm of the insecticide for young children and pregnant women. Whilst there have been improvements, 27.3 % of respondents (all women of reproductive age) in a study in Zambia in 2000 believed that the chemical on the net could be dangerous to the foetus or pregnant woman, whilst in 2004 this proportion was 6.5 %. Misconceptions about malaria can impact on net use; in Nigeria, it was seen that pregnant women who did not have any misconceptions about malaria prevention had 1.56 (*p* = 0.036) the odds of using an ITN [24]. In addition, people would generally not stop using or refuse a net if they did not like its colour (Likert score: −0.62) suggesting that unmet preferences are not a strong inhibitor of net use. The shape of the net, i.e., rectangular compared to conical (which is often said to be easier to hang) was not a problem, with only 2.5 % of those with difficulties stating this as the reason, i.e., it was a problem for 0.1 % of all net recipients from the campaign. A previous study found that conical ITNs were significantly more likely to be used than rectangular ITNs [18]. If net design is a prominent inhibitor of ITN use then investing in medications of ITN at the manufacturer level can be considered [11]. There was also no difference in hanging between polyester and polyethylene (adj OR 1.12, *p* = 0.27) nets in a separate model that included only LLINs, but adjusted for the same factors.

### Treatment effects

Analysis of the treatment effects of BCC outcomes on net use applying models that create counterfactuals from observational data and thereby control for confounding influences, confirms a significant success of BCC-related outcomes increasing net use by 17 %-points by increasing confidence to take action from poor to excellent, or by 15 %-points. Discussing net use in the family had a more limited effect with an 8 %-point increase. An analysis of a specific BCC campaign in the context of ITN mass distribution in Cameroon found an increase in ITN use of overall 6.6 % points using a propensity score matching methodology and a 12.0 % point increase for children under five [9]. Using the same methodology of propensity score matching Boulay and colleagues [20] found a 29.5 % point increase attributable to BCC exposure in Zambia based on Demographic and Health Survey data which reduced to 12.7 % points when a treatment effect model similar to the one used in this study, was used. This suggests that the impact of BCC on ITN use found in Nigeria after adjusting for other contributing factors is in a very similar order of magnitude as in other African countries. It also suggests that other factors than BCC remain important determinant of ITN use and some of these have been highlighted in this study: seasonality, intra-household net ownership and the socio-cultural environment (here represented by the north–south differences).

### Strengths and limitations

The strengths of this analysis is the fact that pooling of ten post-campaign data sets provided a large sample size which allowed detailed analysis of rare events, such as reported difficulties in hanging of nets or net use by specific age and gender groups. Furthermore, these surveys all had a population representative design, equivalent to that of a DHS or MIS and collected sufficiently detailed data on message exposure and particularly on respondents’ KAPs with multiple Likert-scale questions, which proved to be critical to construct composite BCC-related outcome measures. However, a number of limitations need to be kept in mind. First, this analysis was opportunistic in the sense that it relied exclusively on survey and household interview data and did not actively attempt to measure actual exposure to at least the media channels. Such data is available in Nigeria through media marketing agencies, but collecting them was beyond the scope of this secondary data analysis. Second, household interview surveys depend on the quality of the interview responses and are prone to recall bias and misclassification, and poor understanding of the questions by the respondents. These can potentially distort or dilute measured responses and effects. However, the results were consistent across different variables and outcome measures suggesting that these types of errors were minor and did not invalidate the overall picture obtained. Third, the post-campaign surveys were done in different places at different times so that information for variables such as season or regions were not done at the same time, which could have influenced the findings. This was addressed or at least limited by including all relevant variables on time and place in all regression models. Fourth, although the ten surveys (five in the north and five in the south) covered a large part of the country (with the exception of the northeast), they were chosen purposively based on the need and implementation area of specific projects. This implies that the samples were representative for each state but not statistically representative for the country as a whole. This could reduce the external validity (generalizability) of the findings, but only if states not included would be significantly different from other states within the same region. Fifth, while exposure to information through various communication channels was recorded in the surveys as perceived by the respondents, there was no information available at the time of analysis as to the intensity, quality or geographical spread of the BCC interventions. This mapping exercise is currently ongoing and should be considered in the final considerations of the findings. Finally, observational data sets, such as the post-campaign surveys, generally are not ideal to assess the impact of specific interventions as they do not have clearly defined control groups nor allow a before-after assessment in the same population. Therefore, whether a person or household actually was or was not exposed can be influenced by a number of other factors and, similarly the treatment effects can be confounded in observational data. This limitation was addressed by applying appropriate statistical methods that have been developed specifically for this purpose and can largely, but not completely, avoid over- or under-estimation of intervention effects. It must be kept in mind, however, that experimental designs that could more precisely answer the questions of BCC impact on net hanging and use and cover diverse regions and seasons would be prohibitively expensive and unrealistic.

## Conclusions

The findings allow the conclusion that the multi-channel BCC campaign, through the campaign as well as other media, was overall very effective in contributing to an increase in net culture and subsequent net hanging and use, particularly by vulnerable groups. While the data clearly point out that there are differences between eco-climatic or cultural zones, here north and south, independent of seasonal variations, wealth, education etc., they do not suggest that the relationship between increasing confidence to take action for malaria prevention and increasing net use is different in principle between these zones. However, whether more intensive BCC activities are needed in the south, or rather a qualitatively different approach to BCC, cannot be answered from the data available. That would need more qualitative studies of the socio-cultural factors underlying these differences.

## Additional files

10.1186/s12936-016-1463-7 Map of Nigeria with surveyed states and rainfall patterns.
10.1186/s12936-016-1463-7 Details of methods. Providing further details on the analytical approach and the treatment effect model.
10.1186/s12936-016-1463-7 Sources of information by channel and sub-categories. Table of proportions of all households and those exposed to any message that reported hearing information on nets from specific channels.
10.1186/s12936-016-1463-7 Results: attitude, knowledge and perception scores Presents detailed findings from the composite scores analyses.
10.1186/s12936-016-1463-7 Results from regression analysis on action score, discussion of net use and intention to use net. Presents detailed results from three logistic regression models.

## Abbreviations

BCC: behaviour change communication
CI: confidence intervals
DHS: Demographic and Health Survey
IEC: information, education and communication
ITN: insecticide-treated net
IPWRA: inverse-probability weight regression adjustment
KAP: knowledge, attitudes and practices
LLIN: long-lasting insecticidal net
MIS: Malaria Indicator Survey
NMEP: National Malaria Elimination Programme
OR: odds ratio
RMS: research and marketing services
SuNMaP: Support to the National Malaria Programme
PMI: US President’s Malaria Initiative
WHO: World Health Organization

## Footnotes

1 The questionnaire did not allow a measure of multiple exposures to the same information source or message.

2 Single question in the questionnaire: Did you discuss sleeping under the net with your family?

3 Single question in the questionnaire: Do you intend to make sure your family sleeps under the nets every night, most nights, some nights?
