# Supplementary figures and images for: The impact of behaviour change communication on the use of insecticide treated nets: a secondary analysis of ten post-campaign surveys from Nigeria
Source: Malar J. 2016 Aug 19;15:422. doi: 10.1186/s12936-016-1463-7 (PMC4992294; doi:10.1186/s12936-016-1463-7)

Figure 4: Map of Nigeria with surveyed states and rainfall patterns

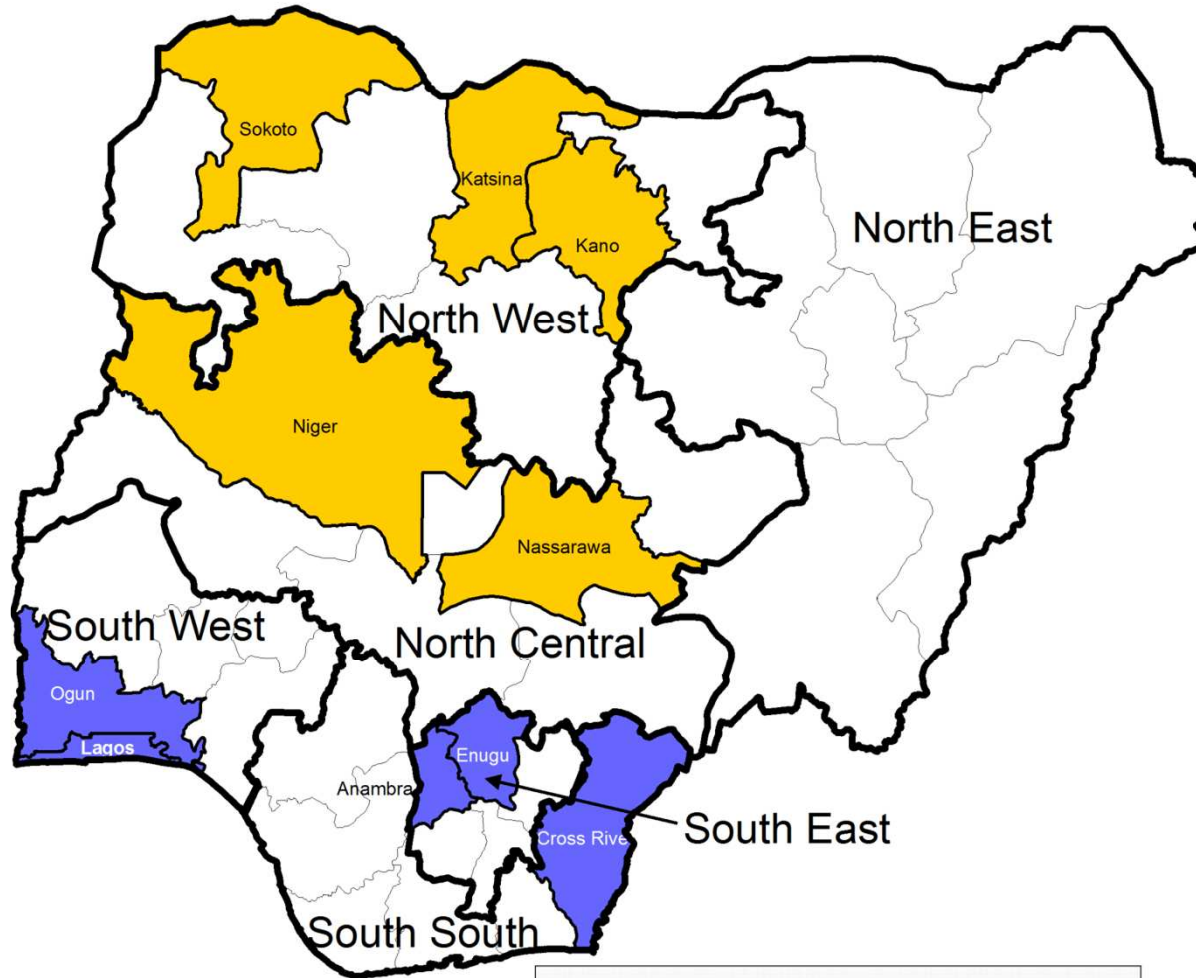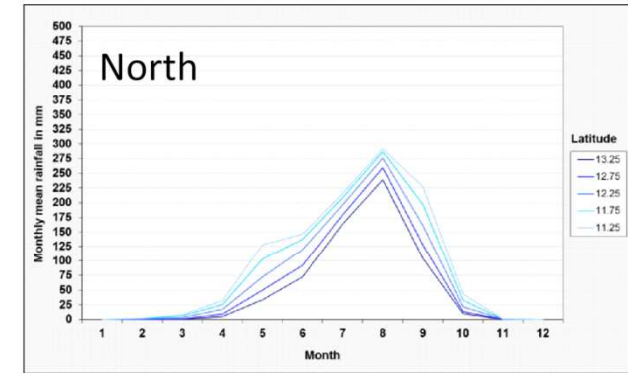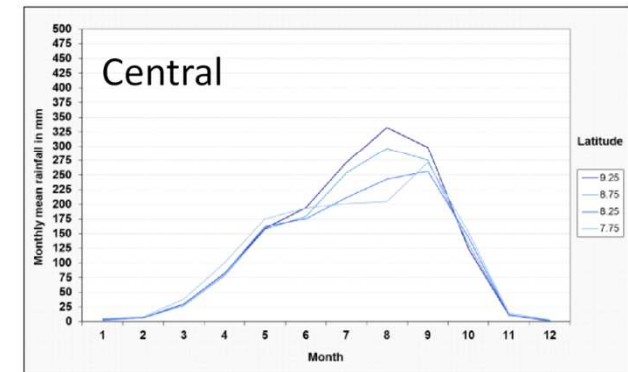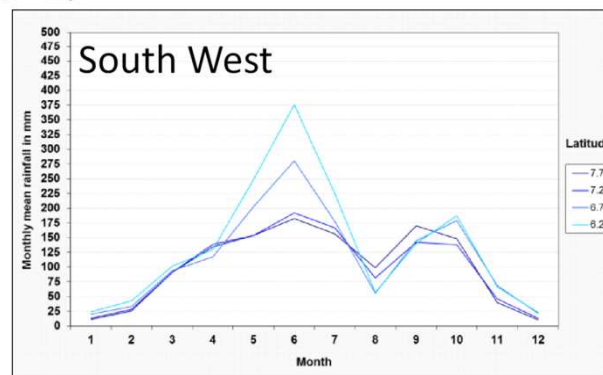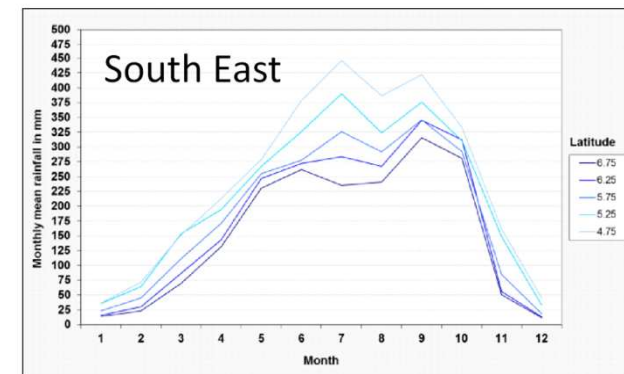

Supplement: Supplementary file 1 — 10.1186/s12936-016-1463-7 Map of Nigeria with surveyed states and rainfall patterns. [file 12936_2016_1463_MOESM1_ESM.pdf]
