# Supplementary material for: The impact of behaviour change communication on the use of insecticide treated nets: a secondary analysis of ten post-campaign surveys from Nigeria
Source: Malar J. 2016 Aug 19;15:422. doi: 10.1186/s12936-016-1463-7 (PMC4992294; doi:10.1186/s12936-016-1463-7)
Supplement: Supplementary file 2 — 10.1186/s12936-016-1463-7 Details of methods. Providing further details on the analytical approach and the treatment effect model. [file 12936_2016_1463_MOESM2_ESM.pdf]

Additional file 2

Details of methods

Analytical approach

The following overall analytical approach was taken to assess the impact of BCC on ITN use:

- First, the exposure to messages about net hanging and use was explored with respect to coverage (any exposure) and intensity (number of information sources[1]) and each communication channel was characterized with respect to its profile in reaching the target population.
- Second, the link between recall of any content and that of specific messages was explored to establish whether there is a dose-response relationship between exposure and recall, i.e. the more exposure the more messages are recalled.
- Third, an association between message recall and specific KAP outcomes relevant to hanging and using of nets is explored. The outcomes were determined by the data available from the surveys and included three measures
    - Confidence in taking action to protect the family with nets (Q58-Q63 in Annex B)
    - Reported discussing of net use within the family[2]
    - Expressed intention to use the nets every night[3]
- These three steps together intend to define BCC exposure dependent outcomes as a composite measure of the BCC effects that can then directly be linked to net hanging and use in uni-variable and multi-variable analysis.
- If BCC mediated effects can be established, the final step is the attempt to quantify the treatment effects from BCC with a quasi-experimental approach that estimates the counterfactuals from within the observational data using treatment effect models (details see "treatment effects" section below).

Treatment effects analysis

The limitation of observational data in the evaluation of the effects of a specific intervention is that for each respondent (here households) only one event can be recorded, either the intervention or the non-intervention (control), but there is no direct comparison of intervention and control possible in the same household. This makes comparisons highly prone to over- or under-estimation due to confounding factors that influence either the probability that a household is exposed or not or the probability that the outcome occurs or both.

However, there are statistical methods that can be used to address the problem. One is the propensity score matching where the probability (propensity score) that a person or household is exposed to the intervention is calculated in a regression model using all relevant covariates and then respondents are matched against the propensity score so that each household with exposure is compared to a household without exposure but with the same probability of being exposed. However, propensity score matching does not include the potential confounders of the outcome measure.

---

[1] The questionnaire did not allow a measure of multiple exposures to the same information source or message
[2] Single question in the questionnaire: Did you discuss sleeping under the net with your family?
[3] Single question in the questionnaire: Do you intend to make sure your family sleeps under the nets every night, most nights, some nights?

The other method is a treatment effect model that combines a model for the outcome with a model for the intervention (treatment) in order to estimate a counterfactual for each observation, i.e. what would have happened if an exposed household would not have been exposed and vice versa, thereby allowing an evaluation of the treatment effect alone. In the case of net use it can be expected from the literature that a number of other factors besides BCC influence the outcome such as rainy season, age and gender of a person etc.
