# Supplementary material for: The impact of behaviour change communication on the use of insecticide treated nets: a secondary analysis of ten post-campaign surveys from Nigeria
Source: Malar J. 2016 Aug 19;15:422. doi: 10.1186/s12936-016-1463-7 (PMC4992294; doi:10.1186/s12936-016-1463-7)
Supplement: Supplementary file 3 — 10.1186/s12936-016-1463-7 Sources of information by channel and sub-categories. Table of proportions of all households and those exposed to any message that reported hearing information on nets from specific channels. [file 12936_2016_1463_MOESM3_ESM.pdf]

Additional file 3

Sources of information by channel and sub-categories

Table 7: Sources of information by major and sub-categories (multiple responses possible)

Information Source	All Households	If any Information
	% (95% CI)	% (95% CI)
**Campaign**	**19.7% (18.0, 21.5)**	**33.7% (30.9, 36.6)**
Leaflet	14.6% (13.1, 16.3)	25.0% (22.5, 27.8)
Team	6.2% (5.2, 7.3)	10.6% (9.0, 12.5)
Distribution Point	0.1% (0.0, 0.2)	0.1% (0.0, 0.3)
**Media**	**21.5% (19.7, 23.5)**	**36.8% (33.9, 39.8)**
Radio	16.4% (14.7, 18.1)	28.0% (25.4, 30.7)
Drama	5.9% (4.8, 7.3)	10.1% (8.1, 12.5)
Press	0.1% (0.1, 0.2)	0.2% (0.1, 0.4)
**Mediators**	**15.7% (14.1, 17.4)**	**26.8% (24.4, 29.4)**
Leaders	9.6% (8.5, 11.0)	16.5% (14.5, 18.6)
Town announcer	6.3% (5.4, 7.5)	10.9% (9.3, 12.8)
Faith based	1.9% (1.5, 2.4)	3.2% (2.5, 4.1)
**Health System**		
Health worker	**18.6% (16.9, 20.5)**	**31.9% (29.2, 34.7)**
**Social network**		
Family or friends	**14.8% (13.3, 16.5)**	**25.4% (23.0, 28.0)**
