# Supplementary material for: The impact of behaviour change communication on the use of insecticide treated nets: a secondary analysis of ten post-campaign surveys from Nigeria
Source: Malar J. 2016 Aug 19;15:422. doi: 10.1186/s12936-016-1463-7 (PMC4992294; doi:10.1186/s12936-016-1463-7)

Additional file 4

Results attitude, knowledge and perception scores

The "ability to take action to protect the family using ITN" was the major measurement of **attitudes** towards net use and data was available for all ten surveys. Likert score questions were used to measure confidence to take action: the following six responses, in order of strongest agreeing response: "I can protect my family from malaria" (Likert score: 1.51), "hang net above all sleeping places" (1.29), "obtain enough nets for all children" (1.23), "sleep under net every night of the year" (0.96) and "get all children to sleep under a net" (0.93). People were least confident that they could "save enough money to obtain enough nets for all children" (0.89). The overall "action" score was positive (1.13, 95%CI 1.10, 1.17). In total, 8.8% did not feel confident to take action (action score <0.0), one in five respondents (19.8%) had a moderately positive attitude (score 0.0-0.9), one in four (25.3%) had a positive attitude (score 1.0-1.4) and almost half (46.0%) a strongly positive attitude towards action (score 1.5-2.0).

Statements on knowledge about ITN and perceptions and beliefs about their use were available for only 4,309 (42%) households from four states (Nasarawa, Enugu, Lagos and Cross River). The Likert score was used to measure **knowledge**, with the following results: "sleeping under a net is the best way to protect from mosquitoes" (1.31), "sleeping under a net is the best way to protect from malaria" (1.22), "dead mosquitoes on the ground is a sign that insecticide is working" (1.19), "new nets protect for several years" (0.83), "insecticide does not harm children" (0.80), "insecticide does not harm pregnant women" (0.78), "some people who sleep under a net still get malaria" (0.08) and "insecticide on nets can be dangerous" (-0.43). Overall, only 7% of households in the four states had poor knowledge and the majority (57%) were scored as good, with a further 30% showing very good and 6% excellent knowledge.

There were no major misconceptions to **perceptions/beliefs**; the following statements were used to measure perceptions and beliefs of ITNS: "nets are difficult to use when warm" (0.76), "it only takes a few months before the net gets too many holes" (-0.14), "nets provide privacy in crowded houses" (-0.29), "many people prefer not to use nets" (-0.38), "expensive nets work better than cheap or free nets" (-0.57), "people don't use nets if they don't' like the colour" (-0.62) and "nets only work over certain beds" (-0.63).

Details of the mean scores for each question are shown in the figures below:

Figure 6: Responses to individual questions regarding action to protect with nets

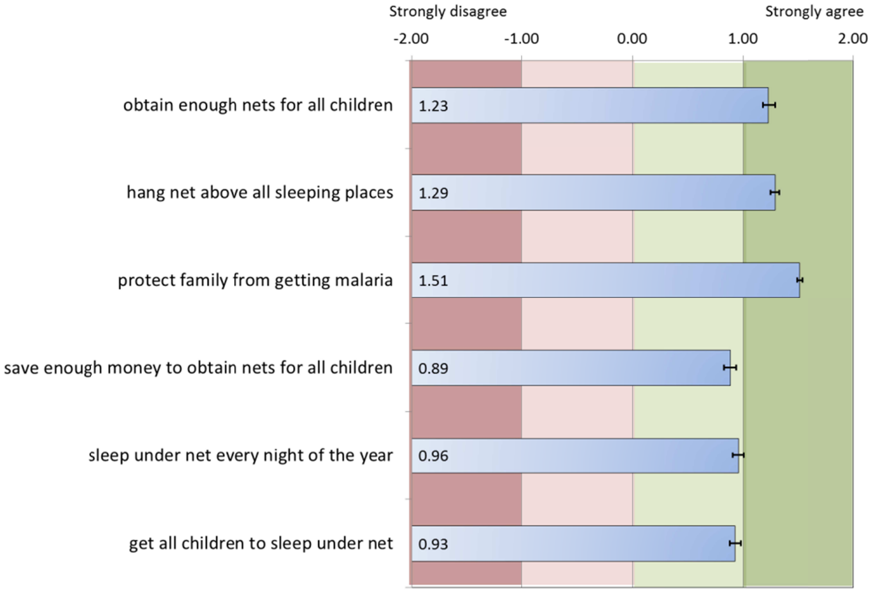

Figure 7: Responses to individual questions regarding knowledge about ITN

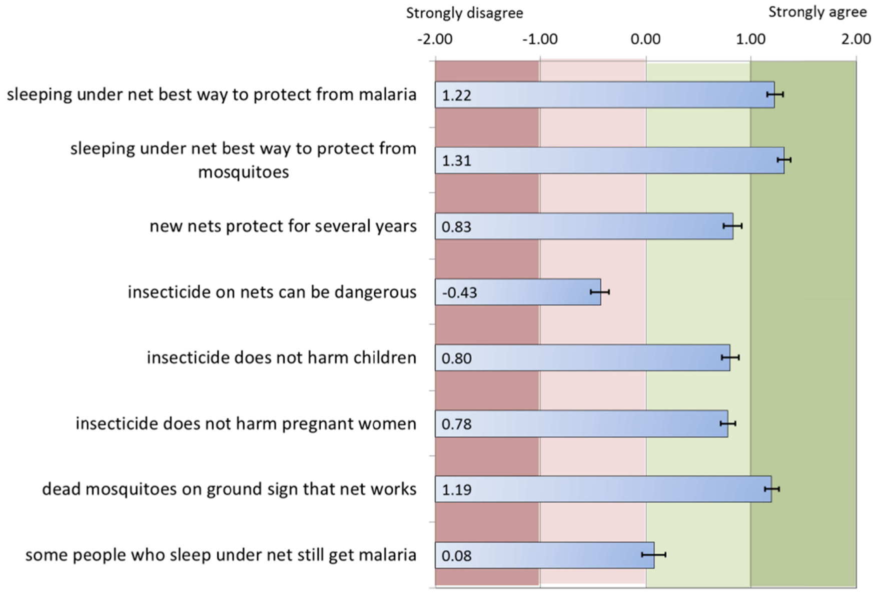

Figure 8: Responses to individual questions regarding perceptions and beliefs about ITN

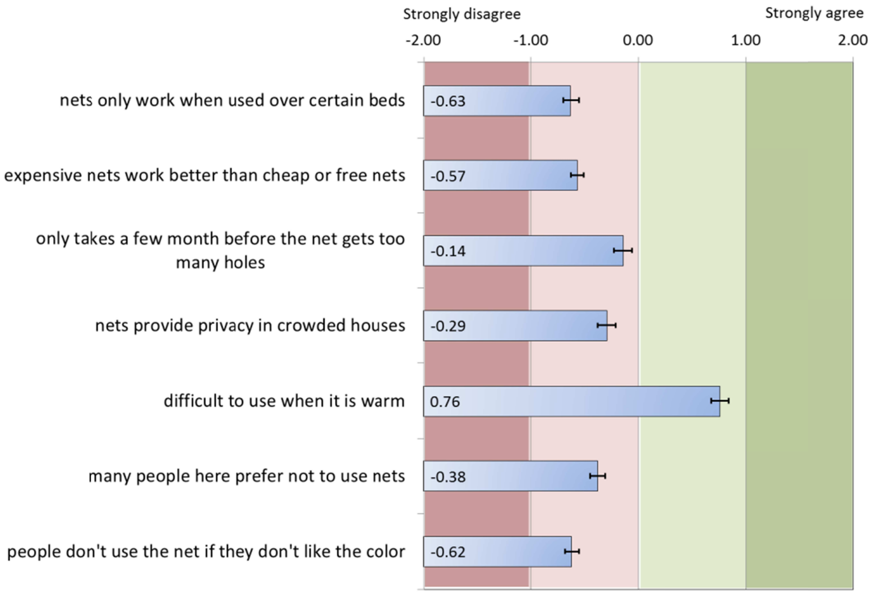

Supplement: Supplementary file 4 — 10.1186/s12936-016-1463-7 Results: attitude, knowledge and perception scores Presents detailed findings from the composite scores analyses. [file 12936_2016_1463_MOESM4_ESM.pdf]
