# Supplementary material for: The impact of behaviour change communication on the use of insecticide treated nets: a secondary analysis of ten post-campaign surveys from Nigeria
Source: Malar J. 2016 Aug 19;15:422. doi: 10.1186/s12936-016-1463-7 (PMC4992294; doi:10.1186/s12936-016-1463-7)
Supplement: Supplementary file 5 — 10.1186/s12936-016-1463-7 Results from regression analysis on action score, discussion of net use and intention to use net. Presents detailed results from three logistic regression models. [file 12936_2016_1463_MOESM5_ESM.pdf]

Additional file 5

Results from regression analysis on action score, discussion of net use and intention to use net

Table 8: Multi-variable linear regression of action score

Explanatory variable	Outcome: action score		
	Coefficient*	95% CI	p-value
Number of messages recalled			
0	ref.	- -	- -
1	0.01	-0.06, 0.07	0.87
2	0.11	0.03, 0.18	0.004
3	0.14	0.06, 0.23	0.001
4	0.25	0.12, 0.38	<0.001
5	0.30	0.12, 0.49	0.001
Time since campaign			
0-3 months	ref.	- -	- -
3-6 months	0.11	-0.03, 0.26	0.12
6-9 months	0.36	0.17, 0.55	<0.001
9-16 months	0.34	0.13, 0.55	0.002
Season			
Dry	ref.	- -	- -
Mid-rain	0.48	0.38, 0.58	<0.001
Late rain	-0.12	-0.26, 0.02	0.096
Wealth quintiles			
Poorest	ref.	- -	- -
Second	0.06	-0.01, 0.12	0.11
Third	0.04	-0.04, 0.12	0.31
Fourth	0.08	0.00, 0.16	0.068
Wealthiest	0.08	-0.01, 0.16	0.084
Education of head			
Non-literate	ref.	- -	- -
Primary	0.04	-0.02, 0.10	0.17
Secondary	0.16	0.10, 0.22	<0.001
Tertiary and higher	0.18	0.09, 0.26	<0.001
Age of head in years			
19-29	ref.	- -	- -
30-59	-0.06	-0.12, -0.01	0.047
60+	-0.14	-0.23, -0.05	0.002
HH owns any mosquito net	0.17	0.10, 0.24	<0.001
HH with 3 or less people & no children	-0.09	-0.16, -0.04	0.001
North vs. South	0.40	0.32, 0.49	<0.001
2009 vs. 2010-12	0.84	0.66, 1.04	<0.001

* describes the change in mean action score induced by covariate

Table 10: Multi-variable logistic regression of discussing net use and intent to use nets

	Outcome 1: discuss net use			Outcome 2: intent to use net every night		
Explanatory variable*	OR	95% CI	p-value	OR	95% CI	p-value
BCC messages recalled						
0	1.00	- -	- -	1.00	- -	- -
1	3.36	2.7, 4.2	<0.001	1.34	0.9, 2.0	0.13
2	4.04	3.1, 5.3	<0.001	1.36	1.0, 1.8	0.033
3	5.14	3.7, 7.2	<0.001	1.63	1.2, 2.3	0.004
4	7.65	3.7, 15.9	<0.001	1.05	0.6, 1.8	0.85
5	10.09	3.6, 28.1	<0.001	0.92	0.5, 1.7	0.78
Confidence to take action						
Poor	1.00	- -	- -	1.00	- -	- -
Good	2.36	1.8, 3.1	<0.001	1.38	1.0, 1.9	0.043
Very good	3.21	2.4 4.3	<0.001	1.79	1.3, 2.4	<0.001
Excellent	4.00	3.0, 5.3	<0.001	4.36	3.2, 5.9	<0.001
Net ownership						
No net	1.00	- -	- -	1.00	- -	- -
Net but not from campaign	4.76	3.4, 6.7	<0.001	1.69	1.0, 2.8	0.038
Campaign net	7.33	5.8, 9.3	<0.001	2.14	1.6, 2.8	<0.001
Season						
Dry	1.00	- -	- -	1.00	- -	- -
Mid-rain	1.65	1.3, 2.2	<0.001	4.79	3.7, 6.2	<0.001
Late-rain	1.21	0.9, 1.6	0.16	3.98	3.0, 5.3	<0.001
Region						
North vs. South	1.65	1.3, 2.1	<0.001	1.45	1.1, 1.9	<0.001
Residence						
Urban vs rural	1.07	0.9, 1.3	0.54	0.69	0.5, 0.9	0.008
Age of head of household						
Age 19-29 vs. 30+	0.75	0.6, 0.9	0.003	1.04	0.9, 1.3	0.66
Education of head of household						
Non-literate vs primary or higher	0.80	0.7, 0.9	0.011	0.70	0.6, 0.8	<0.001
Household size						
HH with 3 or less people & no children	0.73	0.6, 0.9	0.001	0.96	0.8, 1.1	0.61

*The variables time since campaign and year were included in models to adjust for differences in surveys
